# Medium optimization to analyze the protein composition of *Bacillus pumilus* HR10 antagonizing *Sphaeropsis sapinea*

**DOI:** 10.1186/s13568-022-01401-1

**Published:** 2022-05-23

**Authors:** Yun Dai, Ya-Hui Wang, Min Li, Mei-Ling Zhu, Tong-Yue Wen, Xiao-Qin Wu

**Affiliations:** 1 Co-Innovation Center for Sustainable Forestry in Southern China, College of Forestry, Nanjing Forestry University, Nanjing, 210037 Jiangsu China; 2 Jiangsu Key Laboratory for Prevention and Management of Invasive Species, Nanjing Forestry University, Nanjing, 210037 Jiangsu China

**Keywords:** *Sphaeropsis* shoot blight disease of pine, *Bacillus pumilus* HR10, Optimization of fermentation medium, Antagonistic protein

## Abstract

A previous study found that a biocontrol bacterium, *Bacillus pumilus* HR10, inhibited the *Sphaeropsis* shoot blight disease of pine, and the fermentation broth of HR10 strain contained protein antifungal substances. The optimal formulation of the fermentation medium for the antagonistic substance of *B. pumilus* HR10 was finally obtained by single-factor test, Packett-Burman test, steepest ascent test and Box-Behnken Design (BBD) response surface test, and the best formulation of the fermentation medium for the antagonistic substance of *B. pumilus* HR10 was 12 g/L corn meal, 15 g/L beef extract and 13 g/L magnesium sulfate, with a predicted bacterial inhibition rate of 89%. The fermentation filtrate of *B. pumilus* HR10 cultured with the optimized medium formulation was verified to have an inhibition rate of (87.04 ± 3.2) % on the growth of *Sphaeropsis sapinea* by three replicate tests. The antagonistic crude protein of *B. pumilus* HR10 were further isolated and identified using HiTrap Capto Q strong Ion-Exchange Chromatography and LC–MS-MS, and it was speculated that glycoside hydrolase (Ghy), beta-glucanase (Beta), arabinogalactan endonuclease β-1,4-galactanase (Arab), and immunosuppressant A (ImA) are proteins with antagonistic activity against *S. sapinea* in the *B. pumilus* HR10.

**Supplementary Information:**

The online version contains supplementary material available at 10.1186/s13568-022-01401-1.

## Keypoints

- Fermentation medium with high antagonistic activity of HR10 strain was determined.
- Antagonistic crude protein of HR10 strain purified by Ion-Exchange Chromatography.
- Composition of the antagonist protein of HR10 strain was analyzed using LC-MS-MS.

**Supplementary Information:**

The online version contains supplementary material available at 10.1186/s13568-022-01401-1.

## Introduction

At present, among many biological control microorganisms, *Bacillus* spp. has received much attention from researchers because of its high resistance, wide range of habitat and high safety, and plays an important role in the biological control of plant diseases (Berg 2009; Ongena and Jacques 2008). *Bacillus* can control plant diseases through a variety of mechanisms, the most direct way is antagonism. Antagonism refers to the growth process of antifungal substances produced by the bacterium to inhibit the mycelial growth and spore germination of the pathogen, disrupting the normal physiological metabolism of the mycelial cells and the synthesis of cellular inclusions, and eventually leading to the normal growth or even death of the pathogen (Alina et al. 2015; Chen et al. 2020; Perez-Garcia et al. 2011; Sella et al. 2015).

Researchers have identified a variety of antagonistic substances from different biocontrol strains, mainly including two categories: antagonistic substances synthesized by the ribosomal synthesis pathway, including bacteriocins, cell wall degrading enzymes and antimicrobial proteins; and lipopeptide antibiotics synthesized by the non-ribosomal synthesis pathway, including bacilysin, surfactin, iturin, fengycin and kurstakin, etc. (Beneduzi et al. 2012; Cesa-Luna et al. 2020; Dimkic et al. 2022; Fira et al. 2018). The mixture of iturin and surfactant from *Bacillus subtilis* OG strain exerted antagonistic effects by inducing destruction of the cell wall, shrinkage, and leakage of intracellular content in *Xanthomonas campestris* pv. *campestris* and by causing swelling and roughness of the cell surface of *Xanthomonas axonopodis* pv. *citri* (Etchegaray et al. 2008). Fengycin from *Bacillus velezensis* achieved better inhibition of mycelial growth in *Fusarium solani* than chemical fungicides, and its activity remained stable even at high temperatures and low pH values (Wang et al. 2021). *Rahnella aquatilis* JZ-GX1 produces volatile gases that antagonize *Colletotrichum gloeosporioides*, which causes *Liriodendron chinense* × *tulipifera* black spot, by containing 3-methyl-1-butanol and 2-methyl phenyl ether ethyl ether (Kong et al. 2020). Liu et al. (2007) used a DEAE-Sepharose Fast Flow weak ion exchange column to chromatograph the crude protein secreted by *B. subtilis* B-916 strain, and after obtaining an active elution peak, the protein spectrum identified a novel antibacterial protein, Bacisubin, with a molecular weight of 41.900 kDa. The purification procedure of ion-exchange chromatography on DEAE-Sephacel and Sephadex G-100 column chromatography isolated a glycoprotein with a molecular weight of approximately 71.9 kDa from *Paenibacillus polymyxa* Jsa-9 fermentation broth, which exhibited broad-spectrum antibacterial and antifungal activities (Deng et al. 2011).

Pine shoot blight disease is a worldwide disease of pines caused by *Sphaeropsis sapinea* and mainly affects the tops of pines or causes trunk ulcers, resinous and crown and root rot (Cedeno et al. 2001; Stanosz and Kimbler 1997). This disease affects a large number of species, has a large area and is developing rapidly, seriously endangering the health of pine plantations in many countries worldwide (Flowers et al. 2003; Munck et al. 2009). Pine shoot blight disease has caused serious damage and significant losses to pine plantations in many countries around the world. Some chemical control of pine shoot blight disease has been reported, but there are problems with potential environmental pollution and unsustainability with this form of disease control (Fraser et al. 2016; Denise et al. 2018). Although many microbial agents based on *Bacillus* have been developed, the development and utilization of microbial resources for the control of pine shoot blight disease is still seriously inadequate.

In a previous study, the fermentation filtrate of *B. pumilus* HR10 was found to inhibit mycelial growth and spore germination of *S. sapinea*, and the results of the greenhouse test showed that the relative control effect of the pre-sprayed pine seedlings by HR10 strain was up to 90%, and the analysis of the substances in the fermentation filtrate of *B. pumilus* HR10 revealed that the antagonist substances of strain HR10 were protein-like substances precipitated out by 90% saturated ammonium sulfate (Dai et al. 2021). In order to further analyze the antagonistic substances of *B. pumilus* HR10, this experiment firstly optimized the antagonist fermentation medium of HR10 strain by Plackett–Burman (PB) design, steepest ascent test combined with Box-Behnken Design (BBD) response surface methodology, and further identified the antagonistic substances of HR10 strain by acid precipitation method, ammonium sulfate precipitation method and organic solvent precipitation method using the screened efficient antagonist medium, and then identified the antagonistic substances of HR10 strain by chromatography and mass spectrometry, in order to screen the antagonistic protein of HR10 strain and lay the foundation for the work of controlling pine blight disease.

## Materials and methods

### Biological material

*Bacillus pumilus* HR10 was originally isolated from the rhizosphere soil around mycorrhizal seedlings of *Pinus thunbergii* (Sheng et al. 2014), and now it is preserved in the China Center for Type Culture Collection (CCTCC, NO: NO.M2010143)*. Sphaeropsis sapinea* was isolated from infected *P. thunbergii* from Nanjing Forestry University (Dai et al. 2021).

### Screening of key factors in fermentation medium of HR10 strain by single factor experiment

**Screening of basal fermentation medium** The fermentation basal media were No. 1 (glucose 20.0 g/L, soluble starch 20.0 g/L, ammonium chloride 10.0 g/L, peptone 10.0 g/L, magnesium sulfate 1.0 g/L), No. 2 (sucrose 30.0 g/L, ammonium sulfate 10.0 g/L, yeast extract 3.0 g/L, potassium dihydrogen phosphate 0.3 g/L, magnesium sulfate 5.0 g/L),No. 3 (sucrose 16.0 g/L, soybean flour 8.0 g/L, yeast extract 2.5 g/L, potassium dihydrogen phosphate 2.0 g/L, magnesium sulfate 0.5 g/L), No. 4 (glucose 5.0 g/L, yeast extract 2.0 g/L, urea 3.0 g/L, sodium chloride 10.0 g/L), No. 5 (glucose 10.0 g/L, peptone 5.0 g/L, yeast extract 8.0 g/L, potassium chloride 5.0 g/L), No. 6 (corn flour 14.0 g/L, bean cake flour 46.0 g/L, corn steep liquor 3.0 g/L, magnesium sulfate 2.0 g/L, manganese sulfate 0.04 g/L) (Sa 2018), and LB medium (10 g/L tryptone, 5 g/L yeast extract, 5 g/L NaCl) was used as a control. The fermentation sterile filtrate was added to the PDA medium (boiled juice of 200 g/L potato, supplemented with 20.0 g/L glucose, and 15.0 g/L agar) and then inoculated with *S. sapinea* pieces and incubated for 6 days, and the colony diameter of *S. sapinea* was measured to detect the antagonistic *S. sapinea* activity of the fermentation filtrate of HR10 strain.

$$\mathrm{Inhibition\,rate}(\mathrm{\%})=\frac{\mathrm{colony\, diameter\,of\,control}-\mathrm{colony\,diameter\,of\,treatment}}{\mathrm{colony\,diameter\,of\,treatment}}\times 100$$

**Different carbon sources** The antagonistic activity of the fermentation filtrate of HR10 strain against *S. sapinea* was determined by replacing the yeast extract in LB medium with 5 g/L of sucrose, soybean meal, soluble starch, mannitol, glycerol, lactose, corn flour and corn paste, respectively, and using LB medium as the control.

**Different nitrogen sources** The antagonistic activity of the fermentation filtrate of HR10 strain against *S. sapinea* was determined by replacing the tryptone in LB medium with 10 g/L of beef extract, ammonia water, soybean meal, peptone, ammonium nitrate, ammonium nitrite, ammonium sulfate and corn steep liquor, respectively, and using LB medium as the control.

**Different inorganic salts** The antagonistic activity of the fermentation filtrate of HR10 strain against *S. sapinea* was determined by replacing NaCl in LB medium with 10 g/L of potassium dihydrogen phosphate (KH_2_PO_3_), magnesium sulfate (MgSO_4_), manganese sulfate (MnSO_4_), ferrous sulfate (FeSO_4_), calcium chloride (CaCl_2_), potassium chloride (KCl), zinc sulfate (ZnSO_4_), and calcium carbonate (CaCO_3_), respectively, and using LB medium as the control.

### Plackett–Burman (PB) test

The PB experimental design was performed using Minitab 16 software for the five components of the screened media formulations (Wu et al. 2007). Referring to the medium formulations in Table 1, a PB experimental design with N = 12 was selected, with each factor coding level corresponding to the actual level, and the response value was the inhibition rate (%) of the fermentation filtrate of HR10 strain. Each component was taken as high and low level, and the high level was taken as 1.25 –2 times of the low level, and the response value was the fermentation filtrate inhibition rate of HR10 strain.

**Table 1:** Experimental matrix and response values from randomized runs in the Packett-Burman test

Run	Code variable level					Real variable level (g/L)					Inhibition rate (%)
	A	B	C	D	E	Yeast extract	corn flour	beef extract	peptone	MgSO_4_	
1	1	1	1	− 1	1	10	10	15	10	15	86.30
2	− 1	1	− 1	− 1	− 1	5	10	10	10	10	77.50
3	1	1	− 1	1	1	10	10	10	15	15	85.20
4	− 1	1	1	− 1	1	5	10	15	10	15	85.60
5	1	− 1	1	− 1	− 1	10	5	15	10	10	80.50
6	− 1	− 1	− 1	1	1	5	5	10	15	15	76.60
7	1	1	− 1	1	− 1	10	10	10	15	10	81.60
8	− 1	1	1	1	− 1	5	10	15	15	10	81.20
9	1	− 1	− 1	− 1	1	10	5	10	10	15	78.90
10	− 1	− 1	− 1	− 1	− 1	5	5	10	10	10	74.50
11	1	− 1	1	1	− 1	10	5	15	15	10	79.80
12	− 1	− 1	1	1	1	5	5	15	15	15	77.20

### Steepest ascent test and Box-Behnken Design (BBD)

Based on the screened key factors influencing the antagonistic ability of the fermentation medium, the steepest ascent test was conducted so that the obtained key factor concentrations were as close as possible to the response value extreme zone (Sheng et al. 2016). Based on the key factors and levels of the test determined by the steepest ascent test, a three-factor, three-level BBD was designed using Design Expert 12 software to examine and optimize the fermentation medium of HR10 strain (Tak et al. 2017).

### Extraction and purification of antagonistic protein

Ammonium sulfate was added to the fermentation filtrate of HR10 strain to reach a final saturation of 90%, and the precipitate was collected by centrifugation at 10,000 r/min for 30 min at 4 °C for 24 h. The resulting precipitate was dissolved in 5 mL of 10 mmol PBS buffer (pH 7.5) and volume fixed to 20 mL, followed by overnight dialysis. The dialysate was purified using a protein purification system strong Ion-Exchange Chromatography (HiTrap Capto Q), and the column was equilibrated with PBS buffer (pH = 6.0) before loading, and then the fractions were collected by linear gradient elution with PBS buffer (pH 6.0, 0–1 mol/L NaCl) at a flow rate of 1 mL/min (Li et al. 2018). The growth inhibition rate of *S. sapinea* was measured for each fraction of the elution peak, and then SDS–polyacrylamide electrophoresis (SDS-PAGE) was performed on the fraction with higher inhibition rate, as well as the protein concentration and protease activity were determined.

### Determination of protease activity

The protein concentrations of fermentation filtrate of HR10 strain, crude protein precipitated by 90% saturated ammonium sulfate and ion chromatography were determined by the bovine serum protein (BSA) method. Then the protease activity of the fermentation filtrate of HR10 strain, the crude protein precipitated by 90% saturated ammonium sulfate and the protein purified by ion chromatography were determined by the Folin-phenol Reagent Method. The 1% casein solution was mixed with 125 μL of fermentation filtrate of HR10 strain, 90% saturated ammonium sulfate precipitated crude protein or purified protein by ion chromatography, held at 37 °C for 10 min, and 250 μL of trichloroacetic acid was added. The supernatant was extracted and added with 2.5 mL of sodium carbonate solution (0.4 mol/L) and 0.5 mL of Folin-phenol reagent, and the reaction was developed at 37℃ for 20 min, and the absorbance value at 680 nm was detected. The amount of enzyme that produces 1 μg of tyrosine per minute of hydrolysis of casein at 37 ℃ and pH = 7.4 is defined as one viability unit (U/mL). Specific enzyme activity is the viability unit per mg of enzyme protein (U/mg); the purification multiple is the ratio of specific enzyme activity after each purification step to the specific enzyme activity of the fermentation filtrate.

### Identification of antagonistic protein

The identification of protein gel strips is to separate the sample proteins by gel electrophoresis, then obtain the protein gel strips at different positions on the film, extract the peptides after enzymatic digestion, and then uses mass spectrometry to obtain the mass spectrum of the proteins in these gel strips, and finally uses the protein identification software to identify the proteins in the samples. Digestion of 100 µg protein samples using trypsin, followed by freeze-drying of the digested and desalted peptide liquid. The dried peptide samples were reconstituted with mobile phase A (2% acetonitrile, 0.1% formic acid), centrifuged at 20,000 *g* for 10 min, and the supernatant was taken for injection. Separation was performed by Thermo UltiMate 3000 UHPLC. The sample was first enriched in trap column and desalted, and then entered a self-packed C18 column (75 μm internal diameter, 3 μm column size, 25 cm column length) and separated at a flow rate of 300nL/min by the following effective gradient: 0–5 min, 5% mobile phase B (98% acetonitrile, 0.1% formic acid); 5–45 min, mobile phase B linearly increased from 5 to 25%; 45–50 min, mobile phase B increased from 25 to 35%; 50–52 min, mobile phase B rose from 35 to 80%; 52–54 min, 80% mobile phase B; 54–60 min, 5% mobile phase B. The nanoliter liquid phase separation end was directly connected to the mass spectrometer. The peptides separated by liquid phase chromatography were ionized by a nanoESI source and then passed to a tandem mass spectrometer Q-Exactive HF X (Thermo Fisher Scientific, San Jose, CA) for DDA (Data Dependent Acquisition) mode detection. The main parameters were set: ion source voltage was set to 1.9 kV, MS1 scanning range was 350–1500 m/z; resolution was set to 60,000; MS2 starting m/z was fixed at 100; resolution was 15,000. The ion screening conditions for MS2 fragmentation: charge 2^+^ to 6^+^, and the top 30 parent ions with the peak intensity exceeding 10,000. The ion fragmentation mode was HCD, and the fragment ions were detected in Orbitrap. The dynamic exclusion time was set to 30 s. The AGC was set to: MS1 3E6, MS2 1E5.

The protein identification used experimental MS/MS data and aligned them with theoretical MS/MS data from database to obtain results. The whole process started from converting raw MS data into a peak list and then searching matches in the database with Mascot 2.3.02 software. The search results were subject to strict filtering and quality control, and possible protein identifications were produced. Finally, from the final protein identification list, GO functional annotation analysis and iBAQ quantification were performed.

## Results

### Single-factor optimization of fermentation medium for *B. pumilus* HR10

**Fig. 1:**
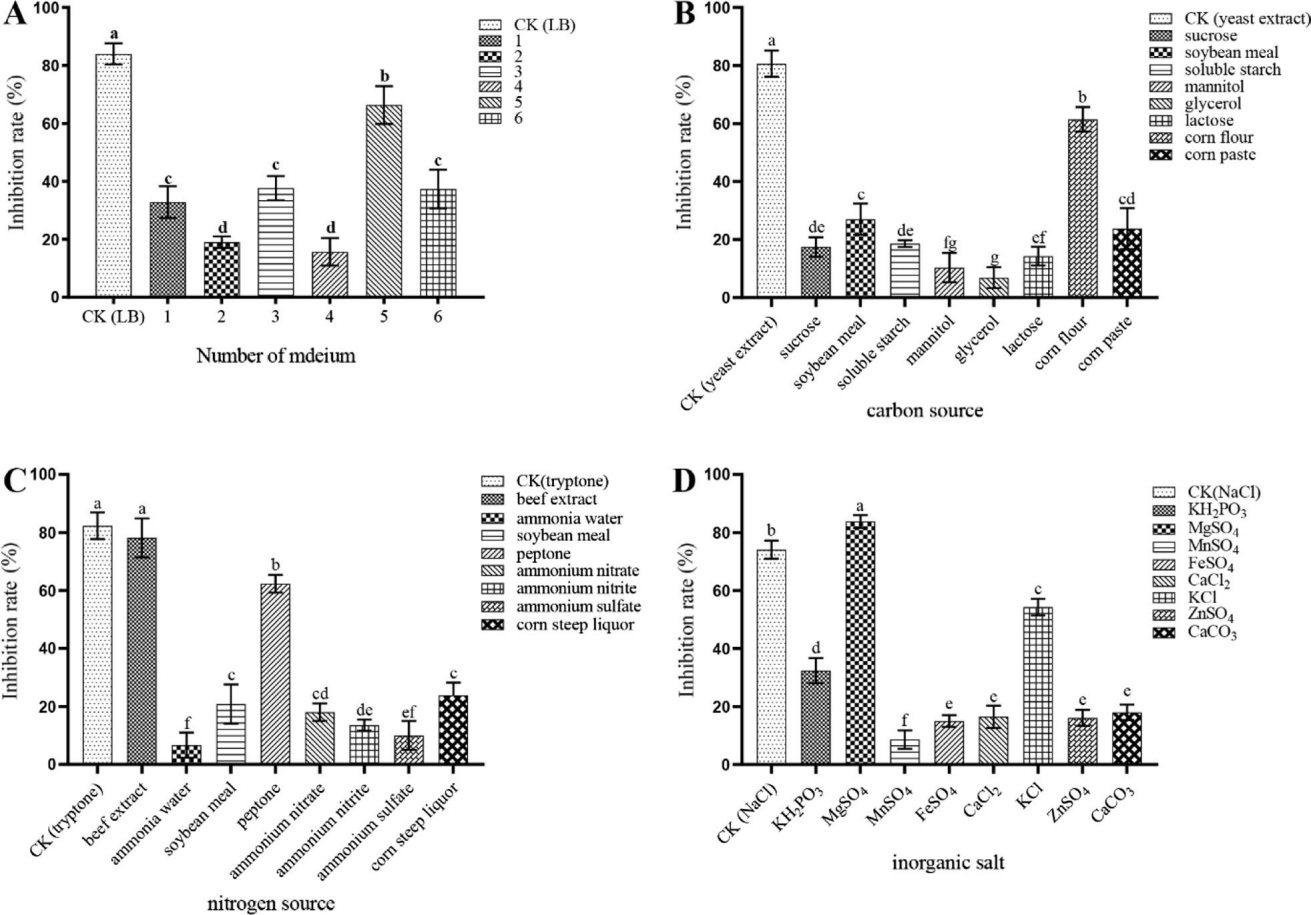
Single-factor test for screening fermentation media of *Bacillus pumilus* HR10 with different basal media (**A**), carbon sources (**B**), nitrogen sources (**C**) and inorganic salts (**D**)

Through the screening of the basal fermentation medium (Fig. 1A), the fermentation filtrate of HR10 strain cultured in LB medium showed the highest inhibition rate of 83% against *S. sapinea*, therefore, the LB medium with yeast extract (5 g/L) as the main carbon source, tryptone (10 g/L) as the main nitrogen source and NaCl (10 g/L) as inorganic salt was selected as the basal medium for subsequent medium optimization. When replacing yeast extract in LB medium with different carbon sources, the inhibition rate showed different degrees of decrease (Fig. 1B), among which the fermentation filtrate of HR10 strain cultured with corn flour as carbon source showed the highest inhibition rate of 64%. Replacing tryptone in the fermentation basal medium with a different nitrogen source showed that replacing tryptone with beef extract showed the best inhibition and was not significantly different from tryptone (Fig. 1C). NaCl in the fermentation basal medium was replaced with different inorganic salts, where the inhibition rate of the fermentation filtrate of HR10 strain with magnesium sulfate as the inorganic salt in the fermentation medium was significantly higher than that of the fermentation filtrate of LB medium (Fig. 1D). Therefore, by changing the carbon and nitrogen sources as well as inorganic salts in the basic fermentation medium formulation and combining with the production cost, yeast extract and corn flour were initially screened as the best carbon source, beef extract and peptone as the best nitrogen source and magnesium sulfate as the best inorganic salt.

### Application of PB for screening important factors affected by antagonism in fermentation media of *B. pumilus* HR10

By applying PB, 12 different formulations were prepared and the experimental runs were carried out based on the experimental matrix of PB, and the observed responses are shown in Table 1. A multiple linear regression model with the response value of the inhibition rate of *S. sapinea* growth by *B. pumilus* HR10 fermentation filtrate was obtained, and the model coefficients and probabilities were statistically analyzed. The inhibition rate of the fermentation broth was described by the regression equation expressed in uncoded units: Inhibition rate (%) = 80.408 + 1.642A + 2.492B + 1.358C−0.14D + 1.225E. The *P*-value of the model was 0.002 and the complex correlation coefficient *R*^2^ = 0.9349. Among the five factors to be investigated, the effect of peptone on the inhibition rate of fermentation filtrate of HR10 strain was not significant, and the remaining four factors were, in descending order of importance, corn flour > yeast extract > beef extract > MgSO_4_, and all of them had positive effects (Table 2). Therefore, corn flour was selected as the optimal carbon source, beef extract as the optimal nitrogen source, and MgSO_4_ as the optimal inorganic salt as the main components of the optimized fermentation medium formulation.

**Table 2:** The importance of each factor for medium optimization

Factors	Coefficient	*T*-value	*P*-value	Significance
A: Yeast extract (g/L)	1.642	4.35	0.005	2
B: Corn flour (g/L)	2.492	6.60	0.001	1
C: Beef extract (g/L)	1.358	3.60	0.011	3
D: Peptone (g/L)	− 0.142	− 0.38	0.720	5
E: MgSO_4_ (g/L)	1.225	3.25	0.018	4

### Optimization of antagonist-producing fermentation media for *B. pumilus* HR10 using the steepest ascent test

In order to further approach the maximum response value region for each main factor for subsequent response surface analysis, the design of the steepest ascent test was performed. Based on the results of PB experiments, it was determined that corn flour, beef extract and MgSO_4_ were the main factors in the optimized *B. pumilus* HR10 fermentation medium, and all three had positive effects. The steepest ascent test design and its response values are shown in Table 3. The results showed that the inhibition rate of *B. pumilus* HR10 fermentation filtrate showed a trend of increasing and then decreasing as the addition of beef extract, corn flour and MgSO_4_ in the medium increased sequentially, indicating that the test design was reliable. When the 4th experimental run, 11 g/L of corn flour, 16 g/L of beef extract and 13 g/L of MgSO_4_, the inhibition rate reached the maximum, which was the region of the maximum response value of the three factors.

**Table 3:** Experimental design and response of the steepest ascent test

Run	Corn flour (g/L)	Beef extract (g/L)	MgSO_4_ (g/L)	Inhibition rate (%)
1	5	10	10	67.80
2	7	12	11	73.40
3	9	14	12	76.80
4	11	16	13	82.00
5	13	18	14	77.00
6	15	20	15	65.00
7	17	22	16	66.50

### Obtaining optimal *B. pumilus* HR10 fermentation medium for protein antagonist production by using BBD

**Fig. 2:**
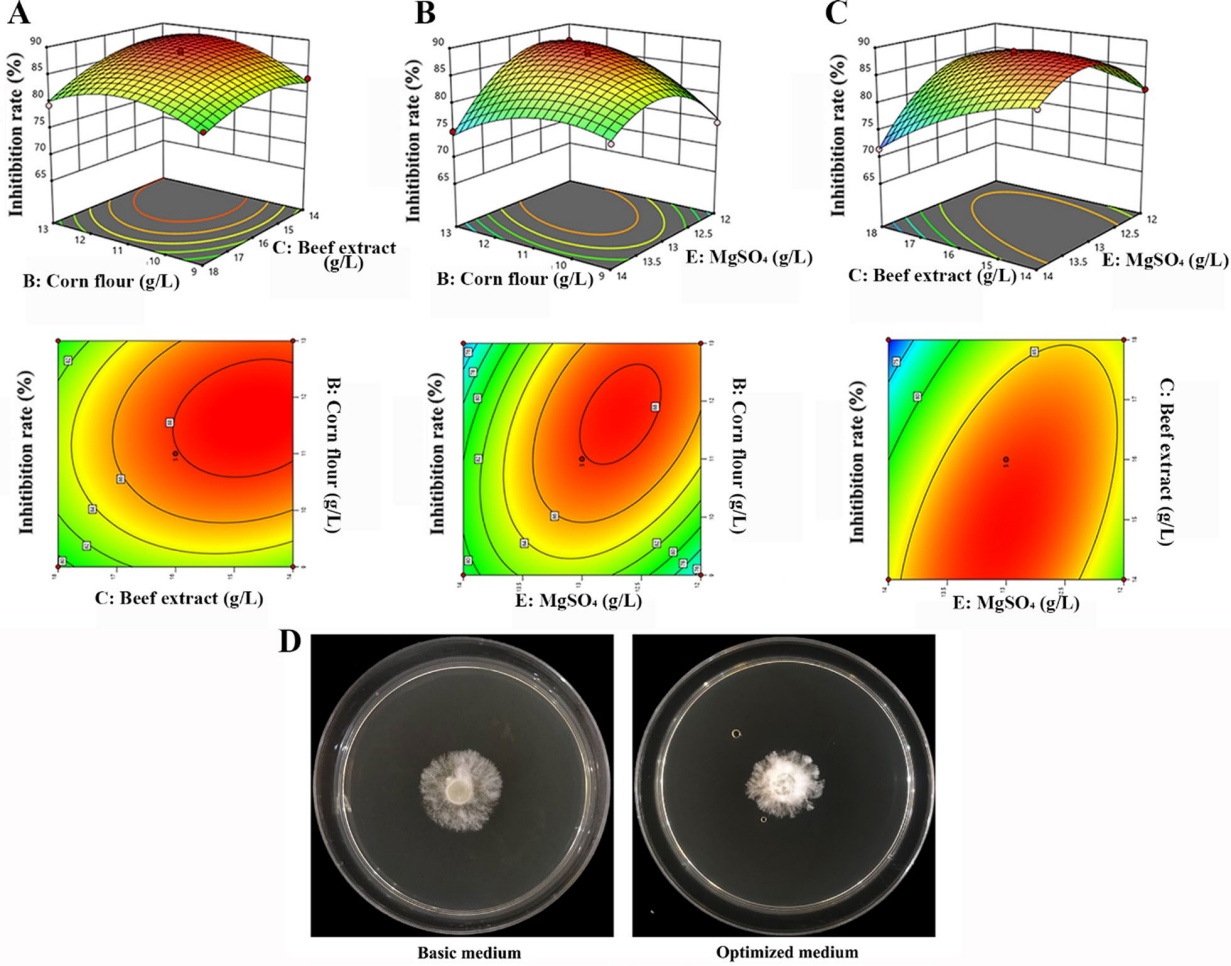
Response surface plots of the effect of various factors on the inhibition rate of *S. sapinea* growth by fermented filtrate of *B. pumilus* HR10 and validation of the optimal formulation. **A** The response surface and contour plots for the effects of corn flour and beef extract on inhibition rate at middle level of MgSO_4_ contents; **B** The response surface and contour plots for the effects of corn flour and MgSO_4_ on inhibition rate at middle level of beef extract contents; **C** The response surface and contour plots for the effects of beef extract and MgSO_4_ on inhibition rate at middle level of corn flour contents; **D** Validation of the optimal formulation for the inhibition rate of *S. sapinea* growth by fermented filtrate of *B. pumilus* HR10

The BBD experimental design and the response values were performed using the inhibition rate of *S. sapinea* growth by the fermentation filtrate of HR10 strain as the response variable with corn flour (B), beef extract (C) and MgSO_4_ (E) as the influencing factors (Table 4). The experimental ANOVA showed that the linear, squared, and interaction effects of the model were significant, and the multivariate correlation coefficient *R*^2^ = 0.989, so the regression equation was a relatively well fit and the predicted values were highly significant in correlation with the experimental values (Table 5, Fig. 2A, B, C). The *P*-value of the Lack of fit was 0.1512, indicating that the model was not significantly out of fit. A regression fit was performed and the quadratic polynomial regression equation obtained: Inhibition rate (%) = 1.53A−2.47B−22.20C−21.13AB−23.88AC−23.77BC−23.59A^2^−22.19B^2^−25.74C^2^.

**Table 4:** Experimental matrix and response values from randomized runs in the Box–Behnken design

Run	Code variable level			Real variable level (g/L)			Inhibition rate (%)
	B	C	E	Corn flour	Beef extract	MgSO_4_	
1	− 1	1	0	9	18	13	79.8
2	0	− 1	− 1	11	14	12	80.9
3	− 1	0	− 1	9	16	12	74.6
4	1	0	− 1	13	16	12	86.7
5	0	0	0	11	16	13	97.4
6	1	− 1	0	13	14	13	86.7
7	0	− 1	1	11	14	14	83.8
8	− 1	− 1	0	9	14	13	82.7
9	1	1	0	13	18	13	79.3
10	0	0	0	11	16	13	88.3
11	0	1	− 1	11	18	12	83.7
12	− 1	0	1	9	16	14	78.2
13	1	0	1	13	16	14	74.8
14	0	0	0	11	16	13	87.9
15	0	1	1	11	18	14	71.5
16	0	0	0	11	16	13	88.7
17	0	0	0	11	16	13	87.2

**Table 5:** Analysis of variance and lack-of-fit tests for the response surface models

Source	Explained sum of squares	DF	Mean square	*F* -value	*P* -value
Model	461.94	9	51.33	70.10	< 0.0001
B: Corn flour	18.61	1	18.61	25.41	0.0015
C: Beef extract	49.00	1	49.00	66.93	< 0.0001
E: MgSO_4_	38.72	1	38.72	52.89	0.0002
B × C	5.06	1	5.06	6.91	0.0339
B × E	60.06	1	60.06	82.04	< 0.0001
C × E	57.00	1	57.00	77.86	< 0.0001
B^2^	54.19	1	54.19	74.02	< 0.0001
C^2^	20.15	1	20.15	27.52	0.0012
E^2^	138.61	1	138.61	189.32	< 0.0001
Residual error Residual	5.12	7	0.7321		
Lack of fit	3.58	3	1.19	3.10	0.1512
Pure error	1.54	4	0.3850		
Cor total	467.06	16			

R^2^ = 0.9890, Adj R^2^ = 0.9749, Predicted R^2^ = 0.8720

Response surface analysis showed that HR10 fermentation filtrate showed the highest inhibition of *S. sapinea* growth at 11.748 g/L for corn flour, 14.917 g/L for beef extract, and 12.860 g/L for magnesium sulfate, with a predicted value of 89%. In order to facilitate the preparation of culture medium, the best formulation of fermentation medium was developed as 12 g/L corn flour, 15 g/L beef extract, and 13 g/L MgSO_4_. The inhibition rates of the three replicate tests were 83.33%, 88.89%, and 88.89%, respectively, with the mean value of (87.04 ± 3.2) %, which was close to the predicted value of 89%, indicating that the model obtained from the response surface test could predict the actual situation. In addition, the inhibition of *S. sapinea* growth by the fermentation filtrate of HR10 strain was increased by 3% after fermentation medium optimization (Fig. 2D), and the cost was lower, indicating that the optimization of the fermentation medium of HR10 antagonist-producing substances by the response surface method achieved more satisfactory results.

### Isolation of antagonistic protein in the fermentation broth of *B. pumilus* HR10 in optimal medium

**Fig. 3:**
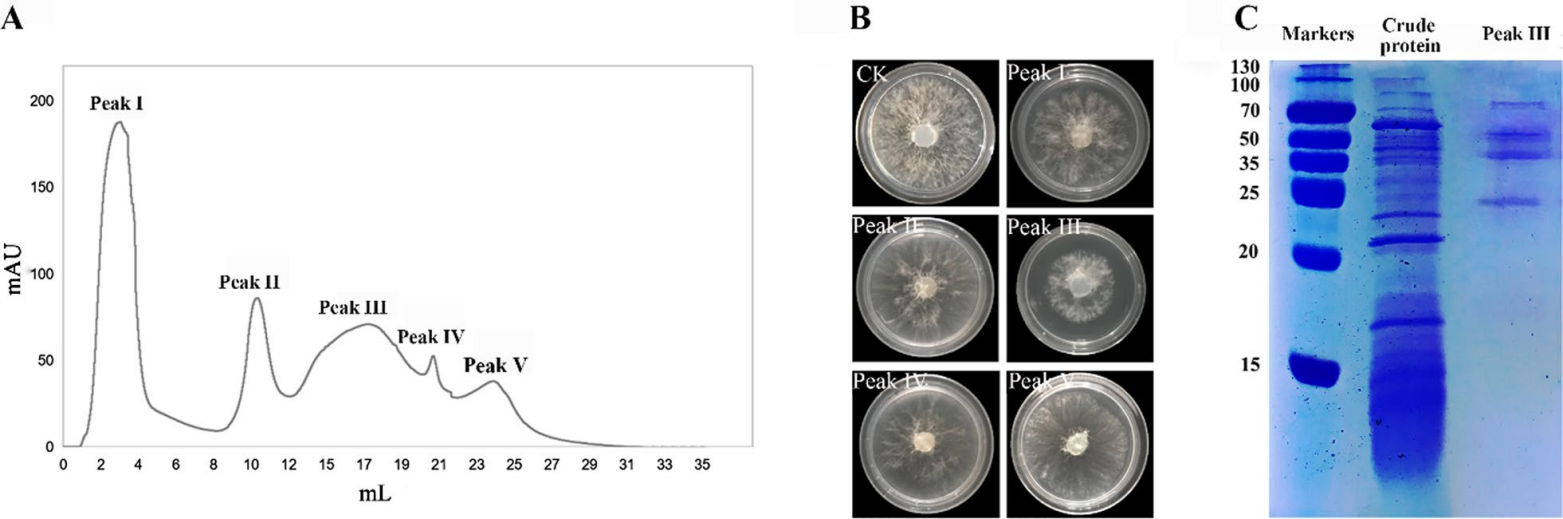
Isolation of antagonistic protein in the fermentation broth of *B. pumilus* HR10. **A** Elution curve of crude protein in *B. pumilus* HR10 fermentation broth by strong Ion-Exchange Chromatography; **B** The antagonistic activity of each elution peak; **C** The SDS-PEGE stained graph of elution peak III

The crude protein obtained from the fermentation filtrate of *B. pumilus* HR10 in optimal medium by 90% saturated ammonium sulfate precipitation was further purified using a HiTrap Capto Q strong Ion-Exchange Chromatography. The resulting elution curves showed a total of 5 peaks (Fig. 3A), and the fractions of the 5 elution peaks were collected separately, desalted and assayed for *S. sapinea* growth inhibition activity. The results showed that peaks I, II, IV and V did not significantly inhibit *S. sapinea* growth, while peak III had inhibitory activity against *S. sapinea* (Fig. 3B). Peak III was detected by SDS-PAGE and showed four clear strips at 20 kDa ~ 100 kDa (Fig. 3C). 500 mL of fermentation filtrate of HR10 strain was purified in two steps, and the final specific activity of antagonistic crude protein reached 283.3 U/mg with a purification multiple of 5.0 (Table 6).

**Table 6:** The purification efficiency of antagonistic protein from *B. pumilus* HR10

Purification of samples	Total protein (mg)	Total enzyme activity (U)	Specific enzyme activity (U/mg)	Purification fold
Fermentation filtrate	38.6	1756	45.5	1.0
Crude protein	25.8	1455	56.4	1.2
Peak III	2.4	680	283.3	5.0

### Identification of antagonistic protein in the fermentation broth of *B. pumilus* HR10

The LC–MS-MS protein spectra of peak III with antagonistic activity obtained by Ion-Exchange Chromatography were analyzed, and a total of 49,368 spectra were obtained, and the number of matched spectra was 1267 after the identification was completed by BLAST, and a total of 220 proteins with 756 peptides were identified (Additional file 1: Table S1). Four proteins were screened by GO functional annotation, namely glycoside hydrolase (Ghy) with cellulase activity whose protein mass was 78,642.23 Da, beta-glucanase (Beta) with hydrolase activity whose protein mass was 27,282.05 Da, immunosuppressant A (ImA) with metallopeptidase activity whose protein mass was 87,240.05 Da, and arabinogalactan endonuclease (Arab) with glucosidase activity whose protein mass was 44,550.25 Da (Table 7). In addition, the scores of all four proteins were greater than 88, indicating that the reliability of the mass spectrometry identification results of all four proteins was high. Therefore, it is hypothesized that the antagonistic protein inhibiting the growth of *S. sapinea* by *B. pumilus* HR10 are one or more of glycoside hydrolase (Ghy), beta-glucanase (Beta), immunosuppressant A (ImA), and arabinogalactan endo-1,4-galactanase (Arab).

**Table 7:** The peptide fragments antagonistic protein of *B. pumilus* HR10 obtained by LC–MS-MS

Protein ID	Name	Match fragment	Protein mass (Da)	Score	GO function	Source strain
tr|W6AP62|W6AP62_9BACI	Glycoside hydrolase (Ghy)	GGLKPSSPTGASDWEK;VAELYYIFAK;YQSSFNEATGK	78,642.23	91.16	Cellulase activity	*Bacillus altitudinis*
tr|A0A0H1RZ89|A0A0H1RZ89_BACPU	Beta -glucanase (Beta)	LSLTSPSYNK;SVQTYGYGLYEVNMKPAK;VQFNYYTNGVGNHEK	27,282.05	92.62	Hydrolase activity	Bacillus *pumilus*
tr|A0A2G8IU99|A0A2G8IU99_BACPU	Immunosuppressant A (ImA)	AWYDIEEDYDYAYVEVR;DPNIDLGEYDQWDR;DVPTYGLSFK;HPGNGFLGVVDADQQVLK;NEAGANEGNGIDGK;NYVNPNNPDAGR;VLVLLVDFK;VPGTEPTGFSPYAK;VWSDDAEGTSTFTFNGFSQSNGK;WSDGSIAATGFQVHDAAFSLNPSTK	87,240.05	88.41	Metallopeptidase activity	
tr|A0A5D4QPP2|A0A5D4QPP2_BACPU	Arabinogalactan endo-β-1,4 -galactanase (Arab)	ALPSLDVFK;AYNGGTNDLNTAIALSK	44,550.25	88.52	Glucosidase activity	

## Discussion

Antagonistic protein secreted by *B. pumilus* HR10 have the potential to antagonize *S. sapinea* causing *Sphaeropsis* shoot blight disease of pine and have high production application value (Dai et al. 2021). In order to maximize the screening of antagonistic protein and to provide data to support the large-scale and low-cost expansion of the *B. pumilus* HR10, this study was conducted to optimize the medium for the production of antagonistic protein by *B. pumilus* HR10. Plackett–Burman test, steepest ascent test and Box-Behnken design are widely used methods for optimizing the fermentation process of culture media (Guo et al. 2010; Yang et al. 2020; Yun et al. 2018). In this experiment, firstly, the LB medium was used as the base medium for single-factor experiments, and five factors, namely yeast extract, corn flour, beef extract, peptone and magnesium sulfate, were initially selected as carbon, nitrogen and inorganic salt sources for further screening. The PB test was then used to analyze the importance of the five factors determined by the single-factor test and to screen the components of the optimal fermentation medium.

It is worth noting that in the single-factor experiment, the inhibition rate of fermentation medium with yeast extract as carbon source for HR10 strain (83%) is much greater than that of carbon source with corn flour (64%), however, the importance of corn flour is greater than that of yeast extract by PB test, indicating that with the increase of corn flour addition, its antagonistic effect on the sterile filtrate of HR10 strain is more significant than that of yeast extract. Usually, in fermentation tests, the production rate and fermentation product yield of strains fermented with yeast extract as raw material are higher than those with corn flour as raw material, yet the cost of yeast flour is much higher than that of corn flour. Therefore, in some fermentation process optimization experiments, in order to ensure the effect of prevention and at the same time reduce the cost, the mixture of yeast extract and corn flour is also used as the carbon source (Cheng et al. 2015; Tu et al. 2015). In the optimization of fermentation medium for HR10 strain, it was found that increasing the content of corn flour could be used to completely replace yeast extract as carbon source, which could also guarantee the biocontrol effect of HR10 strain and reduce the cost to a great extent. This may be due to the fact that the nutrients in corn flour are able to promote the biomass increase of HR10 strain fermentation broth to promote its biocontrol effect. By optimizing the biomass fermentation medium of HR10 strain fermentation broth, Lin et al. (2014) used corn flour (7.296 g/L) as the best carbon source to maximize the biomass of HR10 strain fermentation broth.

In addition, the optimized lower cost MgSO_4_ as inorganic salt can also be a good substitute for NaCl in the basal medium. It has long been demonstrated that Mg^2+^ has an important role in protein synthesis, participating in the synthesis of amino acids, in the transcription and translation of genes, in the synthesis of proteins as structural components of ribosomes, and in the activation of various enzymes (Yamaguchi 2011; Yoon and Warshel 2017). Zhu et al. (2020) investigated the biofilm formation ability of HR10 strain under different ion conditions and found that Na^+^ and Mg^2+^ at low concentrations could promote the biofilm formation of HR10 strain. This can reflect that HR10 strain has a preference for Na^+^ and Mg^2+^ to some extent, which verifies the reliability of using Mg^2+^ instead of Na^+^ as the inorganic salt component of HR10 fermentation medium.

In this experiment, the crude protein of strain HR10 antagonistic to *S. sapinea* was further purified by ion chromatography, and the final purification fivefold resulted in a higher enzymatic activity (283.3 U/mg). Finally, by analyzing the results of LC–MS-MS protein spectrum, it was hypothesized that the antagonistic protein with antagonistic *S. sapinea* in *B. pumilus* HR10 were one or more of Ghy, Beta, ImA and Arab. Ghy is a glycoside hydrolase with cellulase activity, a member of the family of endoglucanases capable of cleaving cellulose or related substrates, and most *Bacillus* spp. are capable of secreting cellulases to degrade the cell wall of pathogenic fungi (Mori et al. 2003; Yuan and Gao 2016). Beta is a β-glucanase that belongs to the glycosyl hydrolase 16 family, and β-glucanases are classified into endo- and exo-types. Endo-β-1,3–1,4 glucanase is widely found in bacterial secretory proteins (Khalid et al. 2021; Qiao et al. 2010; Yano et al. 2006). Yao et al. (2004) performed a series of isolation and purification of the fermentation filtrate of *Pa. polymyxa* WY110 and finally obtained β-1,3–1,4 glucanase, which antagonized *Pyricularia oryze*. The secreted protein produced by *B. velezensis* YB15, β-glucanase, antagonized *Rhizoctonia violacea* with up to a width of 10.6 mm, indicating that the antimicrobial substance of strain YB15 was β-glucanase (Xu et al. 2014). Arab is an arabinogalactan, which also has glucosidase activity (Classen et al. 2011). ImA is a zinc-containing metalloantagonist protein, and ImA of *B. thuringiensis* has been reported to specifically hydrolyze antimicrobial peptides produced by insects and disrupt the immune system of some insects (Kwon and Kim 2008). Although the function of ImA in antagonizing pathogenic fungi is currently unknown, it is speculated that ImA also has some ability to antagonize pathogenic fungi by inhibiting host self-defense responses, as the biotrophs and pathogenic fungi usually do not unilaterally cause damage to the pathogenic fungi during the interaction between the biotrophs and the pathogenic fungi, which often also produce a series of self-defense responses.

In this study, the best formulation of protein antagonist fermentation medium for *B. pumilus* HR10 was 12 g/L corn flour, 15 g/L beef paste and 13 g/L magnesium sulfate by Plackett–Burman test, steepest ascent test and Box-Behnken design, which optimized the formulation of antagonist substance fermentation medium for HR10 strain to some extent and provided data to support the future application of *B. pumilus* HR10 biocontrol agent in production. And then the efficient antagonistic protein against *S. sapinea* was isolated and purified using optimized medium and ion exchange chromatography, and the antagonistic activity was increased from 45.5 U/mg initially to 283.3 U/mg, with a purification multiple of fivefold. The obtained protein was not a single substance, and four proteases with hydrolytic activity were initially postulated, laying the foundation for the development and application of engineered strains for the control of *Sphaeropsis* shoot blight disease of pine.

## Supplementary Information

**Additional file 1:** **Table S1.** All peptide fragments antagonistic protein of *B. pumilus* HR10 obtained by LC-MS- MS.

## Data Availability

All data generated or analyzed during this study are included in this published article.

## References

[CR1] Alina SO, Constantinscu F, Petruta CC (2015). Biodiversity of *Bacillus subtilis* group and beneficial traits of *Bacillus* species useful in plant protection. Rom Biotech Lett.

[CR2] Beneduzi A, Ambrosini A, Passaglia LMP (2012). Plant growth-promoting rhizobacteria (PGPR): their potential as antagonists and biocontrol agents. Genet Mol Biol.

[CR3] Berg G (2009). Plant-microbe interactions promoting plant growth and health: perspectives for controlled use of microorganisms in agriculture. Appl Microbiol Biot.

[CR46] Cedeno L, Carrero C, Franco W, Lezama AT (2001). Sphaeropsis sapinea associated with shoot blight, dieback and canker in trunks, branches and roots of Caribbean pine in Venezuela. Interciencia.

[CR4] Cesa-Luna C, Baez A, Quintero-Hernandez V, de la Cruz-Enriquez J, Castaneda-Antonio MD, Munoz-Rojas J (2020). The importance of aatimicrobial compounds produced by beneficial bacteria on the biocontrol of phytopathogens. Acta Biol Colomb.

[CR5] Chen K, Tian ZH, He H, Long CA, Jiang FT (2020). *Bacillus* species as potential biocontrol agents against citrus diseases. Biol Control.

[CR6] Cheng LK, Wang J, Fu Q, Miao LZ, Yang XY, Li SG, Li F, Shen ZQ (2015). Optimization of carbon and nitrogen sources and substrate feeding strategy to increase the cell density of *Streptococcus suis*. Biotechnol Biotec Eq.

[CR7] Classen B, Gramann C, Goellner E, Blaschek W (2011). Arabinogalactan-proteins from *Echinacea purpurea*: characterization, localization and immunomodulating properties. Planta Med.

[CR8] Dai Y, Wu X-Q, Wang Y-H, Zhu M-L (2021). Biocontrol potential of *Bacillus pumilus* HR10 against *Sphaeropsis* shoot blight disease of pine. Biol Control.

[CR9] Deng Y, Lu Z, Lu F, Wang Y, Bie X (2011). Study on an antimicrobial protein produced by *Paenibacillus polymyxa* JSa-9 isolated from soil. World J Microbiol Biotechnol Adv.

[CR44] Denise R, Smith Glen R, Stanosz (2018). Sublethal effects of the methyl benzimidazole carbamate “fungicide” thiophanate-methyl applied to prevent Diplodia shoot blight of pines. For Chronicle.

[CR10] Dimkic I, Janakiev T, Petrovic M, Degrassi G, Fira D (2022). Plant-associated *Bacillus* and *Pseudomonas* antimicrobial activities in plant disease suppression via biological control mechanisms—a review. Physiol Mol Plant Pathol.

[CR11] Etchegaray A, Bueno C, Melo I, Tsai SM, Fiore M, Silva-Stenico ME, Moraes L, Teschke O (2008). Effect of a highly concentrated lipopeptide extract of *Bacillus subtilis* on fungal and bacterial cells. Arch Microbiol.

[CR12] Fira D, Dimkic I, Beric T, Lozo J, Stankovic S (2018). Biological control of plant pathogens by *Bacillus* species. J Biotechnol.

[CR41] Flowers J, Hartman J, Vaillancourt L (2003). Detection of Latent Sphaeropsis sapinea Infections in Austrian Pine Tissues Using Nested-Polymerase Chain Reaction. Phytopathology.

[CR42] Fraser S, Martin-Garcia J, Perry A, Kabir MS, Owen T, Solla A, Brown AV, Bulman LS, Barnes I, Hale MD, Vasconcelos MW, Lewis KJ, Dogmus-Lehtijarvi HT, Markovskaja S, Woodward S, Bradshaw RE (2016). A review of Pinaceae resistance mechanisms against needle and shoot pathogens with a focus on the Dothistroma-Pinus interaction. For Pathol.

[CR13] Guo WL, Zhang YB, Lu JH, Jiang LY, Teng LR, Wang Y, Liang YC (2010). Optimization of fermentation medium for nisin production from *Lactococcus lactis* subsp *lactis* using response surface methodology (RSM) combined with artificial neural network-genetic algorithm (ANN-GA). Afr J Biotechnol.

[CR14] Khalid A, Ye M, Wei CJ, Dai BH, Yang R, Huang SJ, Wang ZG (2021). Production of beta-glucanase and protease from *Bacillus velezensis* strain isolated from the manure of piglets. Prep Biochem Biotech.

[CR15] Kong WL, Rui L, Ni H, Wu XQ (2020). Antifungal effects of volatile organic compounds produced by *Rahnella aquatilis* JZ-GX1 against *Colletotrichum gloeosporioides* in *Liriodendron chinense* × *tulipifera*. Front Microbiol.

[CR16] Kwon B, Kim Y (2008). Benzylideneacetone, an immunosuppressant, enhances virulence of *Bacillus thuringiensis* against beet armyworm (Lepidoptera : Noctuidae). J Econ Entomol.

[CR17] Li L, Tan J, Chen F, Hao D (2018). Colonization of *Bacillus cereus* NJSZ-13, a species with nematicidal activity in Masson pine (*Pinus massoniana* Lamb.). J Forestry Res.

[CR18] Lin SX, Wu XQ, Ding XL, Sheng JM (2014). Medium ingredient optimization for *Bacillus pumilus* HR10 proliferation and propagation. J Microbiol.

[CR19] Liu YF, Chen ZY, Ng TB, Zhang J, Zhou MG, Song FP, Lu F, Liu YZ (2007). Bacisubin, an antifungal protein with ribonuclease and hemagglutinating activities from *Bacillus subtilis* strain B-916. Peptides.

[CR20] Mori S, Akao S, Nankai H, Hashimoto W, Mikami B, Murata K (2003). A novel member of glycoside hydrolase family 88: overexpression, purification, and characterization of unsaturated beta-glucuronyl hydrolase of *Bacillus* sp GL1. Protein Expres Purif.

[CR43] Munck IA, Smith DR, Sickley T, Stanosz GR (2009). Site-related influences on cone-borne inoculum and asymptomatic persistence of Diplodia shoot blight fungi on or in mature red pines. For Ecol Manage.

[CR21] Ongena M, Jacques P (2008). *Bacillu*s lipopeptides: versatile weapons for plant disease biocontrol. Trends Microbiol.

[CR22] Perez-Garcia A, Romero D, de Vicente A (2011). Plant protection and growth stimulation by microorganisms: biotechnological applications of Bacilli in agriculture. Curr Opin Biotech.

[CR23] Qiao JY, Zhang B, Chen YQ, Cao YH (2010). Codon optimization, expression and characterization of *Bacillus subtilis* MA139 beta-1,3–1,4-glucanase in *Pichia pastoris*. Biologia.

[CR24] Sa R-B (2018) The antimicrobial activity substances and disease prevention mechanism of the endophytic antagonistic bacterium N6–34 from poplar. Dissertation, Shandong Agricultural University

[CR25] Sella S, Vandenberghe LPS, Soccol CR (2015). *Bacillus atrophaeus*: main characteristics and biotechnological applications—a review. Crit Rev Biotechnol.

[CR26] Sheng J, Wu X, Hou L, University NF (2014). Isolating and identifying mycorrhiza helper bacteria from the rhizosphere soil of *Pinus thunbergii* inoculated with *Rhizipogen luteous*. J Northeast for Univ.

[CR27] Sheng WU, Huo GH, Han QC, Long HZ (2016). Optimization of carbon and nitrogen source of basic liquid culture of *Gymnopus* sp. producing anti-fungal activity substance and its effect on green and blue mold of citrus. China Biotechnol.

[CR45] Stanosz GR, Kimbler DL (1997). Shoot Blight of Lodgepole Pine Seedlings in Nebraska Caused by Sphaeropsis sapinea. Plant Dis.

[CR28] Tak JW, Gupta B, Thapa RK, Woo KB, Kim SY, Go TG, Choi Y, Choi JY, Jeong JH, Choi HG, Yong CS, Kim JO (2017). Preparation and optimization of immediate release/sustained release bilayered tablets of loxoprofen using Box-Behnken design. AAPS PharmSciTech.

[CR29] Tu Q, Zhao H, Chen J (2015). Optimization of medium for biological potency of fermentation supernatant of *Paenibacillus brasilensis* YS-1. Minerva Biotecnol.

[CR30] Wang C, Ye X, Ng TB, Zhang W (2021). Study on the biocontrol potential of antifungal peptides produced by *Bacillus velezensis* against *Fusarium solani* that infects the passion fruit passiflora edulis. J Agr Food Chem.

[CR31] Wu QL, Chen T, Gan Y, Chen X, Zhao XM (2007). Optimization of riboflavin production by recombinant *Bacillus subtilis* RH44 using statistical designs. Appl Microbiol Biot.

[CR32] Xu T, Zhu T-H, Li SJ, Qiao TM (2014). Fungus-inhibitory activity and gene cloning of β−glucanase from *Bacillus velezensis* YB15. Chin J Biol Control.

[CR33] Yamaguchi M (2011). Regucalcin and cell regulation: role as a suppressor protein in signal transduction. Mol Cell Biochem.

[CR34] Yang XJ, Guo PP, Li M, Li HL, Hu ZL, Liu XW, Zhang Q (2020). Optimization of culture conditions for amoxicillin degrading bacteria screened from pig manure. Int J Env Res Pub He.

[CR35] Yano S, Wakayama M, Tachiki T (2006). Cloning and expression of an alpha-1,3-glucanase gene from *Bacillus circulans* KA-304: The enzyme participates in protoplast formation of *Schizophyllum commune*. Biosci Biotech Bioch.

[CR36] Yao W-L, Wang Y-S, Han J-G, Li L-N, Song W (2004). Purification and cloning of an antifungal protein from the rice diseases controlling bacterial strain *Paenibacillus polymyxa* WY110. Acta Genet Sin.

[CR37] Yoon H, Warshel A (2017). Simulating the fidelity and the three Mg mechanism of pol and clarifying the validity of transition state theory in enzyme catalysis. Proteins.

[CR38] Yuan YH, Gao MY (2016). Proteomic analysis of a novel *Bacillus* jumbo phage revealing glycoside hydrolase as structural component. Front Microbiol.

[CR39] Yun TY, Feng RJ, Zhou DB, Pan YY, Chen YF, Wang F, Yin LY, Zhang YD, Xie JH (2018). Optimization of fermentation conditions through response surface methodology for enhanced antibacterial metabolite production by Streptomyces sp. 1–14 from cassava rhizosphere. PLoS ONE.

[CR40] Zhu ML, Wu XQ, Wang YH, Dai Y (2020). Role of biofilm formation by *Bacillus pumilus* HR10 in biocontrol against pine seedling damping-off disease caused by *Rhizoctonia solani*. Forests.

